# Editorial: Highlights in skin allergy

**DOI:** 10.3389/falgy.2023.1181405

**Published:** 2023-04-26

**Authors:** Laurent Misery

**Affiliations:** ^1^ Laboratoire Interactions Epithéliums-Neurones, Université de Bretagne Occidentale, Brest, France; ^2^ Service de Dermatologie, Vénérologie et Allergologie, CHU Brest, Brest, France

**Keywords:** allergy, allergology, itch, COVID-19, MRGPRX2, DOCK-8

**Editorial on the Research Topic** Highlights in skin allergy

As an interface organ between the inside and outside of our bodies, skin is an immune organ with many dendritic cells, especially Langerhans cells in the epidermis, and a number of more or less transient immune cells such as lymphocytes, mast cells, eosinophils, neutrophils, or basophils. Consequently, skin allergy is frequently the first manifestation of an allergy. We were proud to welcome four interesting papers presenting research in the field of dermato-allergology.

Bettina Wedi, from Hanover, provided an excellent overview of the contemporary grand challenges and opportunities in the field of skin allergies. In the field of atopic dermatitis, numerous new treatments may allow a choice, according to the endotype and/or inflammatory phase, for each patient in the future. Until now, chronic prurigo treatment was frequently very difficult, but the recent progress in research has opened fascinating new therapeutic options (1). We hope that it could be the same regarding mastocytosis or angioedema without weals. In the field of urticaria, omalizumab is the main therapeutic option after antihistamines, according to the recent guidelines (2), and new drugs are coming. Nonetheless, how it works has not been clarified in detail.

Mas-related G protein–coupled receptor X2 (MRGPRX2) was proved to be the target of many of the agonists associated with a second route, inducing degranulation through the effects of neuropeptides, such as substance P, or numerous drugs, independently of IgE. β-arrestins act as opponents of G proteins and lead to signal termination with or without subsequent internalization. Zhao Wang et al. from Berlin, demonstrated that β-arrestin-2 is chiefly involved in signal termination, whereas β-arrestin-1 exerts its effects on skin mast cells by controlling MRGPRX2 abundance.

The diagnosis of hyperimmunoglobulinemia E syndrome is commonly delayed because patients are initially considered as presenting with atopic dermatitis. Minnie Jacob et al. from Riyadh, performed a proteomic analysis on patients with this syndrome related to dedicator of cytokinesis 8 (DOCK-8) deficiency and atopic patients. They evidenced 24/85 distinct proteins. The use of this technique could help to distinguish between DOCK8 deficiency and atopic dermatitis, prevent complications, and initiate the appropriate treatment early.

The occurrence of COVID-19 has rapidly and deeply modified all medical practices. Isabelle Haddad et al. from Beirut, performed an interesting review on the management of patients with atopic dermatitis or chronic spontaneous urticaria in this context.

Hence, the field of dermato-allergology is experiencing transformations, from basic research to patient management, and it is very interesting to observe them.
